# Recombinant domains III of Tick-Borne Encephalitis Virus envelope protein in combination with dextran and CpGs induce immune response and partial protectiveness against TBE virus infection in mice

**DOI:** 10.1186/s12879-016-1884-5

**Published:** 2016-10-07

**Authors:** Anna S. Ershova, Olga A. Gra, Alexander M. Lyaschuk, Tatyana M. Grunina, Artem P. Tkachuk, Mikhail S. Bartov, Darya M. Savina, Olga V. Sergienko, Zoya M. Galushkina, Vladimir P. Gudov, Liubov I. Kozlovskaya, Ivan S. Kholodilov, Larissa V. Gmyl, Galina G. Karganova, Vladimir G. Lunin, Anna S. Karyagina, Alexander L. Gintsburg

**Affiliations:** 1 Gamaleya Center of Epidemiology and Microbiology, Moscow, 123098 Russia; 2 Institute of Agricultural Biotechnology, Moscow, 127550 Russia; 3 Belozersky Institute of Physico-Chemical Biology, Lomonosov Moscow State University, Moscow, 119992 Russia; 4 Chumakov Institute of poliomyelitis and viral encephalitides, Moscow, 142782 Russia

**Keywords:** Tick-borne encephalitis virus, Envelope protein, Domain III, Dextran-binding domain, CpG oligonucleotides

## Abstract

**Background:**

E protein of tick-borne encephalitis virus (TBEV) and other flaviviruses is located on the surface of the viral particle. Domain III of this protein seems to be a promising component of subunit vaccines for prophylaxis of TBE and kits for diagnostics of TBEV.

**Methods:**

Three variants of recombinant TBEV E protein domain III of European, Siberian and Far Eastern subtypes fused with dextran-binding domain of *Leuconostoc citreum* KM20 were expressed in *E. coli* and purified. The native structure of domain III was confirmed by ELISA antibody kit and sera of patients with tick-borne encephalitis. Immunogenic and protective properties of the preparation comprising these recombinant proteins immobilized on a dextran carrier with CpG oligonucleotides as an adjuvant were investigated on the mice model.

**Results:**

All 3 variants of recombinant proteins immobilized on dextran demonstrate specific interaction with antibodies from the sera of TBE patients. Thus, constructed recombinant proteins seem to be promising for TBE diagnostics. The formulation comprising the 3 variants of recombinant antigens immobilized on dextran and CpG oligonucleotides, induces the production of neutralizing antibodies against TBEV of different subtypes and demonstrates partial protectivity against TBEV infection.

**Conclusions:**

Studied proteins interact with the sera of TBE patients, and, in combination with dextran and CPGs, demonstrate immunogenicity and limited protectivity on mice compared with reference “Tick-E-Vac” vaccine.

**Electronic supplementary material:**

The online version of this article (doi:10.1186/s12879-016-1884-5) contains supplementary material, which is available to authorized users.

## Background

Tick-borne encephalitis (TBE) presents a threat for a public health in different countries [1]. TBEV is divided into 3 subtypes: European, Siberian, and Far-Eastern, and the Siberian subtype is the most widespread in Russia [2], nevertheless TBEV strains of different subtypes are serologically related closely [3]. Vaccination with one of the currently used purified inactivated TBE vaccines derived from cell cultures is the main prophylactic tool. European TBE vaccines are prepared from European TBEV strains, and Russian – from Far-Eastern ones. Long history and wide geography of TBE vaccines and tiny percent of incidence among vaccinated use proves their efficacy regardless the original subtype [4]. Technological process of inactivated vaccine preparation includes accommodation of large amounts of highly neurovirulent virus stock, which complicates the vaccine production site and elaborates biosafety and biosecurity measures. Recombinant protein subunit vaccines do not pose this kind of threat. Additionally, full vaccination scheme includes 3 primary injections with booster re-vaccinations every 3–5 years. Vaccination of the population living in endemic areas, is a burden to the federal budget. Therefore, there is a need in development of new low cost subunit vaccines with a safe production process that could cause prolonged immunity without additional revaccinations.

E protein of TBEV and other flaviviruses is located on the surface of the viral particle. E protein mediates the binding of virus to the host cell receptors and the penetration of virus into the cell [5, 6], and is a main target for host immune system antibodies [7, 8]. E protein ectodomain consists of 3 domains: I, II and III. Antibodies against domain III are found in the sera of patients and laboratory animals after infection or vaccination [9]. TBEV E protein domain III has an Ig-fold structure and can fold independently from the rest of the protein molecule [10]. Domain III sequences are conservative (80 – 95 % amino acid sequence identity) among tick-borne flaviviruses [10, 11]. Above mentioned properties make TBEV E protein domain III a promising component for development of subunit vaccines against 3 TBE subtypes and kits for diagnostics of TBEV [12].

Immunogenic potency of TBEV E protein domain III is significantly lower than potency of the whole virion or soluble E protein [13], however, it can be increased by addition of adjuvants. It has been shown that immunogenic potency of E protein domain III of Dengue virus can be significantly increased by addition of CpG oligonucleotides [14]. Similar effects were shown for the E protein domain III of West Nile virus in combination with CpG oligonucleotides [15] or Freund’s incomplete adjuvant [16].

Immobilization of a protein on the carrier results in a longer circulation of this protein in organism, and as a result, in more intense immune response [14]. On the other hand, immobilization of the protein on the carrier also can make it convenient component of a diagnostic system.

In this work the immunogenic properties and protective efficacy of compositions comprising 3 variants of recombinant protein which includes domain III of TBEV E protein fused with the dextran-binding domain immobilized on dextran and CpG oligonucleotides were studied. We found that all 3 variants of TBEV E protein domain III immobilized on dextran can specifically interact with the sera of patients with TBE. Preparation comprising the recombinant TBEV E protein domain III and CpG oligonucleotides induces the production of neutralizing antibodies, but demonstrates limited protectivity as compared with Tick-E-Vac vaccine.

## Methods

### Cells and viruses

Porcine embryo kidney (PEK) cells (obtained from Mechnikov Moscow Research Institute of vaccines and sera, 1959–1965) were maintained at 37 °C in medium 199 (FSUE IPVE, Russia) supplemented with 5 % fetal bovine serum (Gibco ThermoFisher Scientific, USA).

TBEV strains Absettarov (GenBank ID AF091005), isolated in the Leningrad region in 1951; Vasilchenko (GenBank ID AF069066), isolated in the Novosibirsk region in 1969; Sofjin (GenBank ID KC8506252), isolated in Far East in 1937, were from the laboratory virus collection of the Chumakov Institute of poliomyelitis and viral encephalitides. Viruses were stored as aliquots of 10 % infected mouse brain suspensions or infected PEK cells cultural fluids at -70 °C.

### Reference vaccine

“Tick-E-Vac” tissue culture, purified, concentrated, inactivated, sorbed TBE vaccine (FSUE Manufacture of Bacterial and Viral Preparations of Chumakov Institute of Poliomyelitis & Viral Encephalitides, Russia), from the TBEV strain Sofjin, 0.25 ml suspension for intramuscular injection for vaccination of children from 1 to 16 years of age, lot 005, was used in the present work.

### Sera

Sera from patients with acute TBE were kindly provided by Dr. V.V. Pogodina and sera of healthy donors were from laboratory collection of the Chumakov Institute of poliomyelitis and viral encephalitides. Sera samples were collected at Russian hospitals with informed consent from patients or their legal representatives and transfered to the Chumakov IPVE as a part of routine TBE diagnostics.

Hyperimmune ascites fluid (HIAF) was obtained in mice after 3 immunizations with the TBEV strain Ek-328.

### Selection of amino acid sequences of TBEV E protein domain III and multiple alignment

Complete amino acid sequences of TBEV E protein domain III (397 entries) available on 25th of February, 2014 were selected from RefSeq Data Base using BLAST (e = 1e-63). Multiple alignment (see Additional file 1) was constructed using COBALT tool [17].

### Amino acid sequences of TBEV E protein domain III selected based on analysis of multiple alignment (see Results) for construction of recombinant proteins

DIIIS: TBEV strain Zausaev (Siberian subtype, RefSeq ID: AAO43537):

- GLTYTMCDKTKF***A***WKR***T***PTDSGHDTVVMEV***T***FSGTKPCRIPVRAVAHGSPDVNVAMLITPNPTIENNGGGFIEMQLPPGDNIIYVGELSHQWFQKGSSIG

DIIIE: TBEV strain Absettarov (European subtype, RefSeq ID: AAC2088):

- GLTYTMCDKTKF***T***WKR***A***PTDSGHDTVVMEV***T***FSGTKPCRIPVRAVAHGSPDVNVAMLITPNPTIENNGGGFIEMQLPPGDNIIYVGELSHQWFQKGSSIG

DIIIF: TBEV strain Sofjin (Far Eastern subtype, RefSeq ID: AEP25267):

- GLTYTMCDKTKF***T***WKR***I***PTDSGHDTVVMEV***A***FSGTKPCRIPVRAVAHGSPDVNVAMLMTPNPTIENNGGGFIEMQLPPGDNIIYVGELSHQWFQKGSSIG

Amino acid residues different in genetic subtypes are marked by bold and italic.

### Construction of recombinant plasmids for expression of chimeric TBEV E protein domain III/DBD2 gene

The synthetic genes, corresponding to the sequences of 3 variants of TBEV E protein domain III listed in above section were cloned in the earlier constructed plasmid pL125, which includes dextran binding domain from *Leuconostoc citreum* KM20 and Gly-Ser spacer [18]. As a result, 3 recombinant plasmids (pDBD2-D3S, pDBD2-D3E and pDBD2-D3F) were constructed, each coding for chimeric gene composed of nucleotide sequence of *dbd2* gene, Gly-Ser spacer and nucleotide sequence of 1of 3 variants of TBEV E protein domain III. Molecular mass of recombinant proteins calculated according to amino acid sequence was 28.1 kDa.

### Expression procedure

The producing strains *E. coli* M15 [Rep4, pDBD2-D3S], *E. coli* M15 [Rep4, pDBD2-D3E] and *E. coli* M15 [Rep4, pDBD2-D3F] were grown in liquid LB medium with kanamycin (25 μg/mL) and ampicillin (150 μg/mL) using 0.2 mM of isopropyl-β-D-thiogalactoside (IPTG) for induction of protein synthesis. Biomass yield was approximately 5 g/L.

### Isolation and purification of recombinant proteins

Protein purification was performed using Sephadex G200 (ChemBioMed, Russia) chromatography with urea gradient elution and dialysis against PBS buffer at 25 °C overnight. Purity of DBD2-D3S, DBD2-D3E, DBD2-D3F antigen samples was higher than 95 %, as was determined by sodium dodecyl sulfate-gel electrophoresis on 12 % polyacrylamide gels [19].

### Immobilization of proteins on dextran

Protein samples were diluted to 0.25 mg/ml of PBS, equal volume of 100 mg/ml suspension of Dextran 500 (Pharmacosmos, Denmark) was added, and the samples were incubated at +25 °C for 1 hour with constant mixing.

### ELISA

Several modifications of ELISA were used during the present work:

To evaluate the ability of the protein preparations to interact with the anti-TBEV antibodies we used 1) “VectoTBE-Antigen” (Vector Best, Russia) according to the manufacturer’s protocol; and 2) ELISA with standard technique by the following scheme: 1st layer – investigated protein preparation, 2nd layer – sera of TBE patients, 3rd layer – HRP-conjugated anti-human antibodies (Fermentas ThermoFisher Scientific, USA); sera of non-vaccinated donors from non-endemic territories was used as a negative control. Antibody titers in the sera of TBE patients were determined using “VectoTBE-IgG” (VectorBest, Russia) according to the manufacturer’s protocol.

To evaluate the antibody titers in immunized mice sera we used 3) ELISA performed using standard technique by the following scheme: 1st layer – antigen (AG), 2nd layer – dilutions of analyzed sera, 3rd layer – HRP-conjugated anti-mouse antibodies (Fermentas ThermoFisher Scientific, USA). AG was prepared from concentrated cell culture supernatant as described earlier [20]. Normal antigen from non-infected cells was used as a negative control. AG and normal AG were equilibrated by protein content.

### Sodium dodecyl sulfate-polyacrylamide gel electrophoresis and Western blot analysis

The protein samples were separated by sodium dodecyl sulfate-polyacrylamide gel electrophoresis (SDS-PAGE) in 12 % PAAG in Protean II EF cell (Bio-Rad, Russia). Separated protein bands were transferred onto a PVDF-membrane (GE Healthcare, USA). The membrane was blocked with 5 % skim milk in PBS overnight at 4 °C. TBEV proteins were detected using sera of TBE patients or control sera as a primary antibody and HRP-conjugated goat anti-human antibodies (Fermentas ThermoFisher Scientific, USA) as a secondary antibody dissolved in 5 % skim milk in PBS with 0.05 % Tween-20. The bands were visualized by reaction with DAB substrate.

### Fifty percent plaque reduction neutralization test (PRNT50)

PRNT50 was performed as described earlier [21]. Briefly, sequential dilutions of sera were prepared in 199 medium on Earle solution with addition of 2 % FBS (Gibco ThermoFisher Scientific, USA). Equal volume of virus suspension, containing 40−50 PFU, were added to each sera dilution. Virus + sera were incubated at 37 °C for 1 h. Then, virus + sera suspensions were added to PEK cells, and incubated at 37 °C for 1 h with gentle shaking for virus adsorption. Then, cells were overlaid with 5 ml of 1 % bactoagar (Difco, USA) on Earle solution containing 7.5 % FBS and 0.015 % neutral red. After incubation at 37 °C on day 7 plaques were counted. Every experiment included appropriate controls – negative and positive sera with known antibodies titer. The antibodies titer was calculated according to modified Reed-and-Muench method.

### Immunogenic potency in mice

Recombinant proteins were combined into following preparations:

- **3DIII + Dex**: Combination of equimolar quantities of 3 recombinant proteins DBD2-D3S, DBD2-D3E and DBD2-D3F (containing 20 μg of each antigenic domain (D3S, D3E, and D3F) immobilized on 25 mg of Dextran 500 (Pharmacosmos, Denmark).
- **3DIII + AD:** Combination of 3DIII + Dex with CpG-oligonucleotides. Thionilated oligonucleotides CPG B-class specific for mouse TLR9 were synthesized on a 12 column DNA synthesizer Polygen (Polygen GmbH, Germany) using modified protocols with tetraethylthiuram disulfide (TEDT) as sulfurizing reagent [22]. S-thionilated product was purified by reverse phase HPLC (Gilson, USA) using XBridge OST C-18 2.5 μm (4.6 x 50 mm) column (Waters, USA). Mobile phase: A: 0.1 M TEAA, B: Acetonitrile/0.1 M TEAA, 20/80 (v/v) and gradient: 35 − 65 % B in 24 min (7–13 % ACN, 0.25 % ACN per minute), desalted by NAP-10 (GE Healthcare, USA) and lyophilized (Labconco, USA).

BALB/c mice of 22–24 g (Stolbovaya branch of Scientific Centre of biomedical technologies, Moscow region, Russia) were randomized by weight into groups of 10 mice and were immunized subcutaneously (s/c) 3 times with studied preparations (0.5 ml, containing 20 μg of each recombinant protein (D3S, D3E, and D3F), 25 mg of Dextran 500 and 0.25 mg of each CpG (ODN 1585 and ODN 1826)) with 7 days period or 2 times with Tick-E-Vac (0.2 human dose in 0.5 ml) with 7 days period. Control group was injected 3 times with 0.5 ml of physiological saline with 7 days period. Sera were collected 1–4 weeks after the first vaccination.

### Protective efficacy in mice

BALB/c mice of 12–14 g (Stolbovaya branch of Scientific Centre of biomedical technologies, Moscow region, Russia) were randomized by weight into groups of 10 mice and s/c immunized several times (for details see Results) with 0.5 ml of sequential dilutions of studied protein preparations or “Tick-E-Vac” with 7 day period between vaccinations. Control animals were injected with 0.5 ml of physiological saline. Mice were intraperitoneally (i/p) challenged with 200LD_50_ TBEV strain Vasilchenko (0.3 ml) 7 day after the last vaccination. Mice were observed for clinical symptoms and weighted every day for 21 day after the challenge. Mice were assumed as ill if showing clinical symptoms (generalized intoxication, paresis, paralysis) or if losing weight 1.5 g or more per 3 days or longer.

Three mice from each group were bled 1 day prior and 1 day after the challenge. Sera were stored at -80 °C until use.

Procedures on animals were performed according to Directive 2010/63/EU and Appendix A to the European Convention ETS No. 123.

### Statistical analysis

Exact Fisher’s test was used for comparison of survival in the groups of mice. *P* < 0.05 was considered to indicate a statistically significant result.

Log-Rank test was used for the statistical analysis of both survival and illness curves. *P* < 0.01 was considered to indicate a statistically significant result.

## Results

### Estimation of TBEV E protein domain III variability

To select sequences of TBEV E protein domain III, which could cause immunity to most TBEV strains, we evaluated a variety of known amino acid sequences and its possible impact on domain III interaction with neutralizing antibodies.

Ecker and co-authors [23] have shown that amino acid residues at positions 313, 317, 331, and 349 differ in the 3 main TBEV subtypes. Our analysis of 398 available up to date amino acid sequences of TBEV E protein domain III showed that these sequences are highly conservative (>96 % similarity). Meanwhile amino acid residues at position 349 are not specific for any of TBEV subtypes, and the amino acid residues at position 313, 317 and 331 are mainly different for different subtypes of TBEV (see Table 1).

**Table 1:** Representation of amino acid residues in three positions of different TBEV subtypes E protein

Amino acid residue and its position in the E protein			Number of strains of different TBEV subtypes^a^				Total
313	317	331	E	FE	S	ND	
A	T	T			67	19	86
T	T	T	1		28	7	36
T	V	T	3			2	5
A	I	T			2	2	4
T	I	T			2		2
T	I	A		54	1	56^b^	111
T	A	A				1	1
T	T	A		4		5^c^	9
T	A	S	2			6	8
T	A	T	55			80	135
Total number of strains			61	58	100	179	397

^a)^ *E* is European subtype, *FE* is Far-Eastern subtype, *S* is Siberian subtype, *ND* is “not determined”, e.g. the subtype is not specified in amino acid sequence annotation and in the corresponding article. The most common variants are shown in bold, genotypes are listed according to the sequence authors

^b)^Strain 886-84, which was annotated as genotype 5 in [25], belongs to this group

^c)^Strain 178-79, which was annotated as genotype 4 in [25], belongs to this grou

According to combinations of amino acid sequences in 313, 317 and 331 positions the Siberian subtype splits into 2 groups that correspond to Asian (A-T-T) and European (T-T-T) topovariants defined by Karan and co-authors [24].

Data presented in Table 1 demonstrate that according to combinations of amino acid residues at positions 313, 317 and 331 the majority (92 %) of the known TBEV E protein sequences can be attributed to 1 of 3 known TBEV subtypes.

**Fig. 1:**
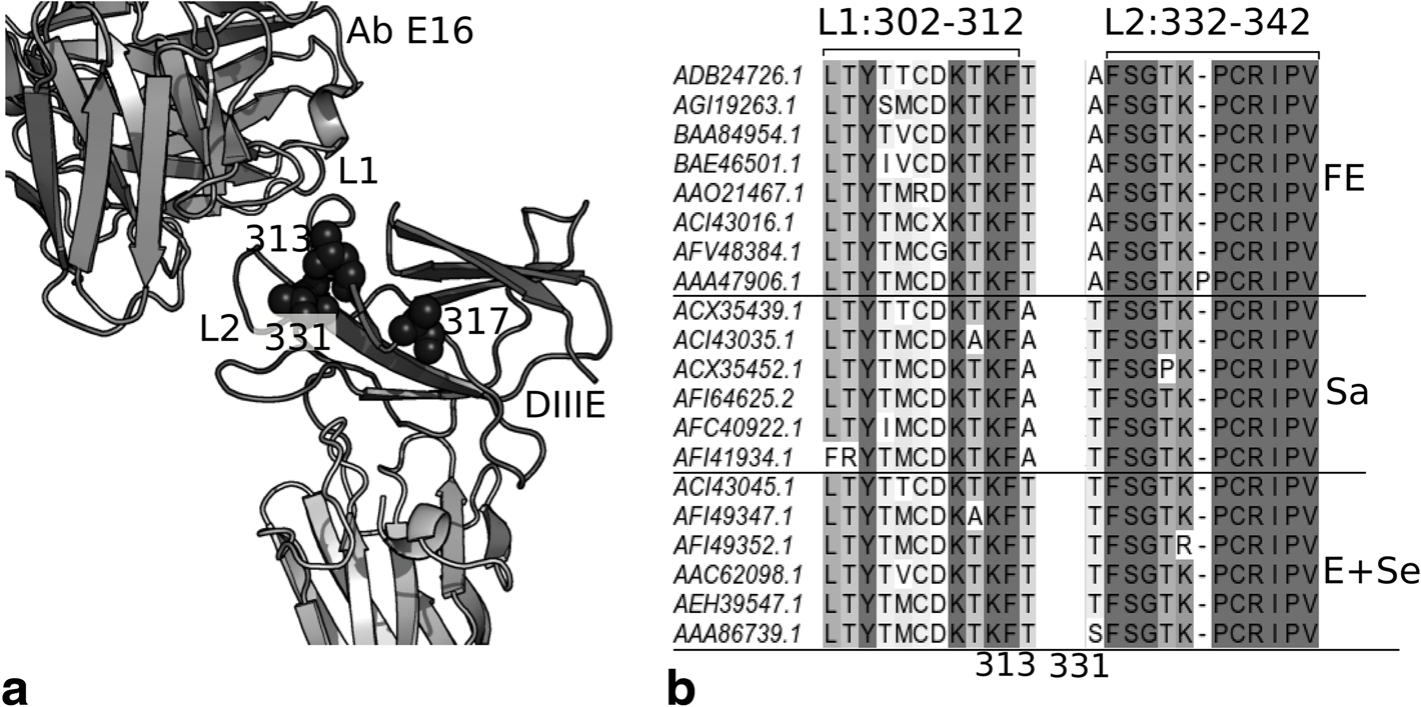
Cartoon representation of spatial structure (**a**) and alignment of amino acid sequences (**b**) of E protein domain III regions involved in the interaction with neutralizing antibodies. **a**. Superimposition of TBEV E protein domain III structure from 1svb PDB entry (DIIIE) with structure of West Nile Virus E protein domain III (is not shown) in complex with neutralizing E16 antibody Fab (1ztx) (Ab E16). Fragment of neutralizing E16 antibody is shown in gray, TBEV E protein domain III is shown in dark gray, the rest part of E protein is shown in light gray, amino acid residues 313, 317 and 331 are shown by black spheres. **b**. Alignment of all different variants of L1 and L2 loops and adjacent 313 and 331 residues. Alignment is highlighted by gray scale fill according to percent of identity. Numbers of amino acid residues forming L1 and L2 loops are designated above alignment (numbering is according to 1svb structure), 313 and 331 residues are designated below alignment. TBEV subtypes are designated on the right as follows: FE is Far-Eastern, Sa is Siberian (Asian topovariant), Se is Siberian (European topovariant), E is European

To evaluate the potential involvement of these amino acid residues in the interaction of TBEV E protein domain III with neutralizing antibodies we analyzed the contact area in spatial structure of complex presented in Fig. 1a. All 3 residues at positions 313, 317 and 331 are located on the protein surface and thus can be accessible for interaction with antibodies. However, in the structure of the virion different regions of domain III can have different accessibility to antibodies. The most well characterized are surface epitopes of E protein domain III of West Nile virus [26–28]. It was shown that neutralizing antibodies to E protein domain III (murine and human) primarily interact with the region of DIII-lr (DIII lateral surface), formed by L1 and L2 loops (see Fig. 1a) [26, 27]. Thus, the sequences of L1 and L2 loops may be of special interest for selection of sequences of antigenic determinants for cloning.

However, as it is seen in Fig. 1b, both loops are highly conservative. The sequence LTYTMCDKTKF in the L1 loop is typical for 92 % of 398 analyzed sequences, the sequence FSGPK-PCRIPV in the L2 loop is typical for 98 % of 398 analyzed sequences. Therefore, the most common variants of these sequences were selected for cloning.

In complex with neutralizing antibody all 3 different amino acid residues in various TBEV subtypes, especially residues at positions 313 and 331, that are directly adjoint to the L1 and L2 loops, can influence the interaction of neutralizing antibodies with E protein domain III (see Fig. 1a). Thus, we selected all 3 variants of TBEV E-protein domain III corresponding to European (T-A-T), Siberian (Asian topovariant) (A-T-T) and Far-Eastern (T-I-A) subtypes for cloning. It should be noted that the sequence of European subtype (T-A-T) with threonine residues in 313 and 331 positions also covers the European topovariant of the Siberian subtype.

### Cloning, expression and purification of recombinant TBEV E protein domain III variants

**Fig. 2:**
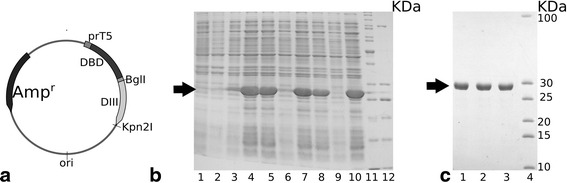
Scheme of recombinant plasmids and analysis of recombinant genes’ expression and proteins purification in 12 % SDS-PAGE (Laemmli, 1970). **a.** Recombinant plasmids pDBD2-DIII. **b**, **c**. Coomassie blue-stained 12 % SDS/PAGE gels of cell extracts (**b**) and purified proteins (**c**). Target proteins are marked by black arrows. **b**. Lanes 1, 3, 6, 9 – extracts of bacterial cells before IPTG induction: *E. coli* M15 [pRep4], *E. coli* M15 [pRep4, pDBD2-D3S], *E. coli* M15 [pRep4, pDBD2-D3E], *E. coli* M15 [pRep4, pDBD2-D3F]. Lanes 4, 5 – cell extracts of *E. coli* M15 [pRep4, pDBD2-D3S], 7, 8 – of *E. coli* M15 [pRep4, pDBD2-D3E], 10 – of *E. coli* M15 [pRep4, pDBD2-D3F] after 2 h IPTG induction. Lane 11 - page ruler unstained molecular mass marker («Fermentas», Lithuania). **c**. Coomassie blue-stained 12 % SDS/PAGE gels of purified recombinant proteins DBD2-D3S (line 1), DBD2-D3E (line 2) and DBD2-D3F (line 3). Line 4 – molecular mass marker Thermo Scientific PageRuler Unstained Low Range Protein Ladder Part No. 26632

Three recombinant plasmids: pDBD2-D3S, pDBD2-D3E, and pDBD2-D3F containing chimeric genes composed of dextran binding domain DBD2 from *Leuconostoc citreum* KM20 (*DBD2*), nucleotide sequence coding for 10 amino acid residues spacer including 5 Gly-Ser repeats, and nucleotide sequence of 1 of variant of TBEV E protein domain III: of Siberian, European or Far-Eastern subtypes, were constructed (Fig. 2a).

Production of recombinant DBD2-D3S, DBD2-D3E and DBD2-D3F proteins determined by electrophoresis was about 20–30 % of total cell protein (Fig. 2b). After purification recombinant proteins were electrophoretically homogenous and were stored as 2 mg/ml solutions (Fig. 2c). For immunological properties study the proteins were immobilized on dextran (see Material and Methods section).

### Evaluation of ability of recombinant preparations to interact with anti-TBEV antibodies

Firstly, preparations of recombinant proteins DBD2-D3S, DBD2-D3E and DBD2-D3F, immobilized on dextran, were investigated as antigen in “VectoTBE-Antigen” ELISA kit. All preparations with protein concentrations 5–20 μg/ml showed ability to interact with monoclonal anti-TBEV antibodies from the kit.

Secondly, we performed ELISA using recombinant proteins DBD2-D3S, DBD2-D3E and DBD2-D3F, immobilized on dextran, as first layer sorbed on the ELISA plate against sera of TBE patients from different territories with different TBEV subtypes prevalent (positive for antibodies against TBEV according to “VectoTBE-IgG” ELISA kit) and healthy people (negative for anti-TBEV antibodies). Thirdly, recombinant proteins DBD2-D3S, DBD2-D3E and DBD2-D3F, immobilized on dextran, were analyzed in PAGE-Western-blot (WB) with the same sera.

Dextran-binding domain itself was used as a negative control in WB; murine TBEV HIAF was used as a positive control in WB and revealed specific band of all studied recombinant proteins, but not DBD (See Additional file 2: Table S1).

Recombinant proteins DBD2-D3S, DBD2-D3E and DBD2-D3F, immobilized on dextran, did not interact with the sera of healthy donors in both ELISA and WB. DBD2 did not interact with any sera in WB. Recombinant proteins interacted with most of the TBE patients sera in ELISA and revealed target band of 28kD in WB.

Therefore, recombinant proteins DBD2-D3S, DBD2-D3E and DBD2-D3F, immobilized on dextran, can specifically interact with antibodies against TBEV.

### Immunogenic potency on mice

Mice were immunized 3 times with studied preparations (3DIII + Dex and 3DIII + AD) or 2 times with reference vaccine “Tick-E-Vac” with 7 days period. Control group was injected 3 times with saline with 7 days period. Sera were collected 1–4 weeks after the first vaccination and analyzed in ELISA individually and in PRNT50 against strains Absettarov, Vasilchenko, Sofjin of 3 TBEV subtypes in pools. Data are presented in Table 2.

**Table 2:** Antibody titers in sera of mice, immunized with studied preparations and the “Tick-E-Vac”

Preparation	ELISA^a^	PRNT50 TBEV strain (subtype)		
		Vasilchenko (Siberian)	Sofjin (Far-Eastern)	Absettarov (European)
1 week after the 1st immunization				
3DIII + Dex	1:10	1:10	1:20	<1:10
3DIII + AD	<1:10	<1:10	1:20	<1:10
Tick-E-Vac	<1:10	<1:10	1:10	<1:10
1 week after the 2nd immunization				
3DIII + Dex	1:70	<1:10	<1:10	<1:10
3DIII + AD	1:500	<1:10	1:30	1:30
Tick-E-Vac	n/s^b^	1:200	1:250	1:70
1 week after the 3d immunization for 3DIII + Dex and 3DIII + AD; 2 weeks after the 2nd immunization for Tick-E-Vac				
3DIII + Dex	1:3000	1:10	1:25	<1:10
3DIII + AD	1:2500	1:20	1:20	<1:10
Tick-E-Vac	n/s	1:20	1:170	1:30
2 weeks after the 3d immunization for 3DIII + Dex and 3DIII + AD; 3 weeks after the 2nd immunization for Tick-E-Vac				
3DIII + Dex	1:4000	1:10	1:20	<1:10
3DIII + AD	1:16000	1:20	1:40	<1:10
Tick-E-Vac	n/s	1:20	1:90	1:30

^a^ELISA was performed by the scheme 3) (see Materials and Methods)

^b^n/s – non-specific interaction. Samples of sera of mice immunized with “Tick-E-Vac” showed high optical density in ELISA with both viral AG and negative control AG, thus the interaction was not determined

According to ELISA antibody titer in mice sera increased in later terms after the vaccinations with both preparations, 3DIII + Dex and 3DIII + AD, which was expected.

Starting from the first week after 2 immunizations antibodies induced by both recombinant protein preparations and “Tick-E-Vac” were detected in both tests. “Tick-E-Vac” caused neutralizing antibodies in higher titers and against strains of different subtypes. Recombinant protein preparations caused significant rise of neutralizing antibodies against strains of Siberian or Far-Eastern subtypes, but antibodies against strain Absettarov of European TBEV subtype rise on the second week, and then decrease to undetectable levels. Nevertheless, 3DIII + AD caused faster and more effective antibody response then 3DIII + Dex. Protein preparation 3DIII + AD was chosen for protective efficacy study in mice.

### Protective efficacy in mice

We evaluated protective efficacy of studied preparation 3DIII + AD against TBEV strain Vasilchenko, as a representative of the most abundant in Russia Siberian subtype, in mice compared to reference vaccine “Tick-E-Vac” according to 2 schemes: standard and prime-boost. In standard scheme mice were immunized 3 times with 3DIII + AD with 7 days period and challenged 7 days after the last vaccination. In prime-boost scheme mice were immunized first with “Tick-E-Vac” and then 2 times with 3DIII + AD with 7 days period and challenged 7 days after the last vaccination. Reference vaccination of mice was performed 2 times with “Tick-E-Vac” with 7 days period and challenged 7 days after the last vaccination. To determine the minimal immunization dose groups of mice were vaccinated with series of preparations dilutions (1, 1/10. 1/32, 1/100, 1/320). Control group was injected 3 times with saline with 7 days period and challenged 7 days after the last injection. Mice were observed for clinical symptoms and weighed 21 days after the challenge. Sera from 3 mice of each group were collected right before and 24 h after the challenge. Sera pools were studied in ELISA and PRNT50 against TBEV strains Vasilchenko, Absettarov and Sofjin. Experimental schemes and test results are summarized in Table 3.

**Table 3:** Protective efficacy of 3DIII + AD and the “Tick-E-Vac” vaccine on mice upon standard and prime-boost schemes

Preparation	N mice	Day 0 1st dose	Preparation	Day 7 2nd dose	Day 14 3^d^ dose	Day 21^b^				Day 42 N mice	
						ELISA	PRNT50 TBEV Vas	PRNT50 TBEV Abs	PRNT50 TBEV Sof	Survived	Healthy
Standard scheme											
3DIII + AD	10	+^a^	3DIII + AD	+	+	1:4000	<1:10	1:50	<1:10	5^c^	0
Prime-boost scheme											
Tick-E-Vac	10	+	3DIII + AD	+	+	1:3000	1:20	1:250	1:50	10^c^	6
Reference scheme											
Tick-E-Vac	10	–	Tick-E-Vac	+	+	1:1500	1:80	1:100	1:250	10^c^	10
Control											
Saline	10	+	Saline	+	+	<1:500	<1:10	<1:10	<1:10	0	0

Mice were challenged with the TBEV strain Vasilchenko (Vas). Antibody titers against TBEV strains Vasilchenko, Absettarov (Abs) and Sofjin (Sof) were measured

^a^+ mice were vaccinated; – mice were not vaccinated

^b^mice were intraperitoneally (i/p) challenged with 200LD_50_ TBEV strain Vasilchenko

^c^ *p* < 0.05 compared to the control group (exact Fisher test)

**Fig. 3:**
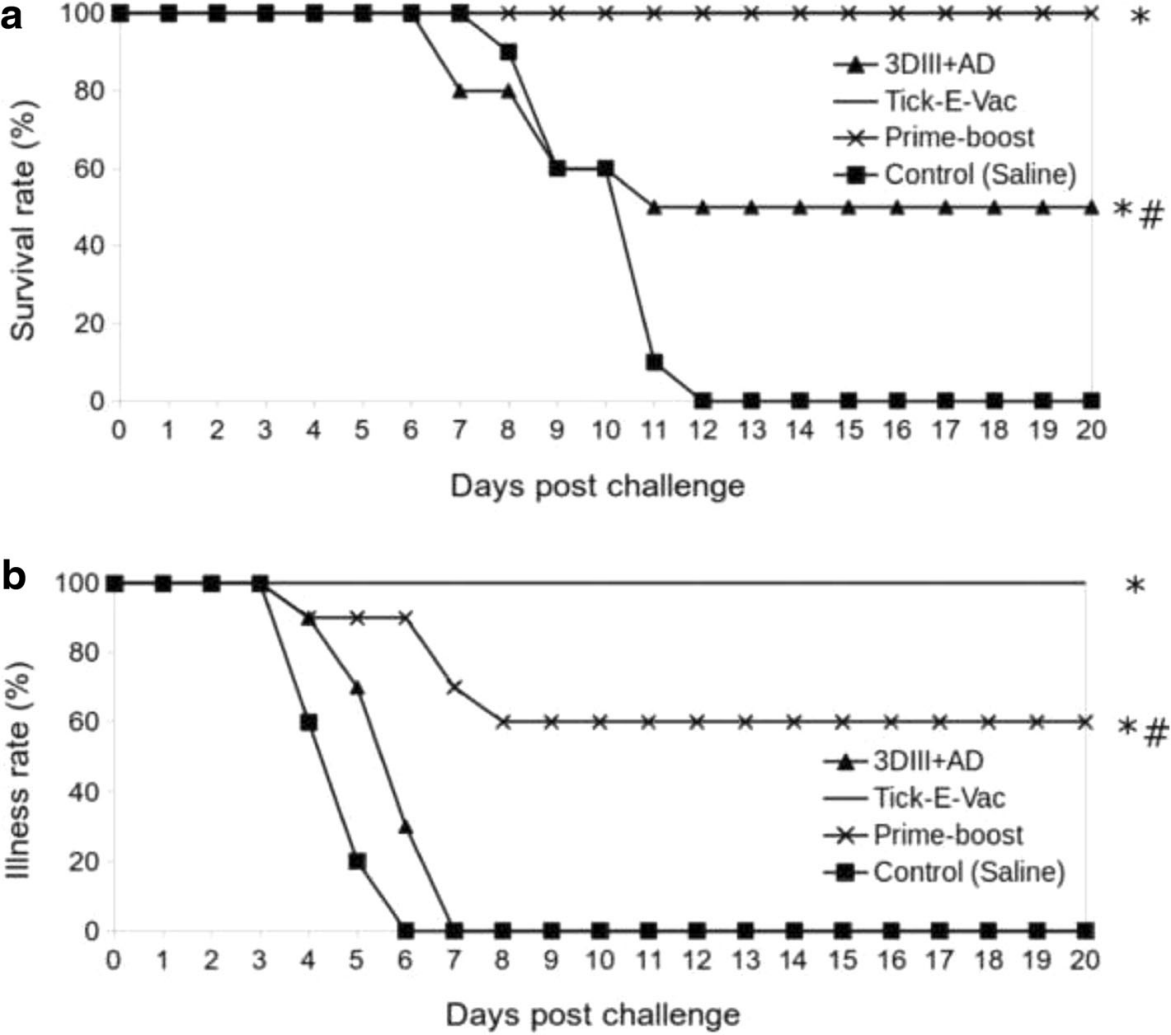
Survival (**a**) and morbidity (**b**) curves. Tick-E-Vac means group of mice (*n* = 10) immunized with Tick-E-Vac by standard scheme; 3DIII + AD means group of mice (*n* = 10) immunized with 3DIII + AD by standard scheme, Prime-boost means group of mice (*n* = 10) immunized with Tick-E-Vac and 3DIII + AD by prime-boost scheme. Group of mice (*n* = 10) immunized by saline was used as a control. Mice were assumed as ill if showing clinical symptoms (generalized intoxication, paresis, paralysis) or if losing weight 1.5 g or more per 3 days or longer. Statistical significance was determined by Log-rank test. * *p* < 0.01 compared with a control (saline), # *p* < 0.01 compared with Tick-E-Vac

With standard scheme 1 dose of 3DIII + AD protected 50 % mice from 100LD50 of TBEV strain Vasilchenko (see Fig. 3a). However, all survived mice showed clinical symptoms of infection (see Fig. 3b, Additional file 2: Table S2). One dose of reference vaccine Tick-E-Vac showed 100 % protectivity, with no mice showing TBE clinical symptoms. With prime-boost scheme combined use of “Tick-E-Vac” and 3DIII + AD provided survival of 100 % mice, although 40 % mice in this scheme showed mild symptoms of infection (see Fig. 3, Additional file 2: Table S2).

Vaccination with preparation dilutions allowed to determine the minimal immunization dose. MID for 3DIII + AD was 0.5 ml (3*20 μg of the recombinant proteins) and for “Tick-E-Vac” – 0.003 ml.

All preparations caused antibody response. Neutralizing antibody titers differed depending on the scheme and TBEV serotype of challenging virus. The highest titers showed prime-boost scheme against TBEV strain Absettarov of European subtype.

## Discussion

Analysis of currently available amino acid sequences of TBEV E protein domain III showed that 92 % of the sequences belonging to 1 of 3 subtypes: European, Siberian or Far Eastern, varied in 3 positions 313, 317 and 331. 8 % of TBEV E protein domain III sequences cannot be attributed to any of the known subtypes according to these 3 diagnostic positions. There is a possibility that the polymorphisms in these positions are the result of recombination between TBE subtypes [29, 30]. The amino acid residues at 313, 317 and 331 positions are on the surface of TBEV E protein domain III (see Fig. 2a), and can contribute to its interaction with the neutralizing antibodies. Therefore, to study the immunogenic potency of the recombinant TBEV E protein domain III we selected 3 variants of amino acid sequences corresponding to the 3 most common subtypes of TBEV. Similarity of the antigenic properties of recombinant TBEV E protein domain III synthesized in *E. coli* to native TBEV E protein domain III structure was showed by ELISA with a set of antibodies to TBEV, as well as by Western-blot analysis demonstrating the interaction of recombinant proteins immobilized on dextran with TBE patients sera. Thus, the constructed recombinant antigens seem to be promising in terms of the development of diagnostic kits for TBE.

Also we demonstrate that immunization with a preparation containing TBEV E protein domain III induces the formation of antibodies against TBEV in titers dependent on the adjuvant composition. On the fourth week after immunization with recombinant antigens with addition of both dextran adjuvant and CpG oligonucleotides the titer of antibodies was 4 times higher than after immunization with a preparation containing only dextran (Table 2).

The neutralization test (PRNT50) with 3 strains representing different TBEV subtypes showed increase in neutralizing antibodies titers after immunization with TBEV E protein domain III over time (see Table 3). Nevertheless, the maximum serum dilution that neutralizes 50 % of the virus obtained after immunization with a preparation containing TBEV E protein domain III in all studied periods was lower than the one obtained after immunization with a Tick-E-Vac vaccine containing inactivated TBEV.

Data on PRNT50 are in good agreement with the literature data on the immunogenic potency of domain III of E glycoprotein of Dengue and West Nile flaviviruses. Chiang and co-authors [14] studied the immunogenic properties of genetically engineered vaccine for prevention of Dengue fever. The investigated candidate vaccine contained recombinant Dengue virus E protein domain III as antigen and CpG oligonucleotides as adjuvant. 6 weeks after the first immunization of BALB/c mice with this preparation (30 μg of Dengue virus E protein domain III in 1 dose) mean titer of serum neutralizing 50 % of the virus was 1:30. In another work 8 weeks after immunization of mice with preparation containing West Nile virus E protein domain III serum titer neutralizing 50 % of the virus was 1:35 [13]. These results are similar to our data on neutralizing activity of the mice serum (see Table 3).

Protective properties of our preparation are also comparable with the literature data [16, 31], but lower than of the whole-virion vaccine.

Therefore, the resulting formulation based on 3 variants of recombinant TBEV E protein domain III immobilized on dextran and CpG oligonucleotides (3DIII + AD) possesses a certain immunogenic potential in comparison to formulation without CpG (3DIII + Dex), see Table 2. It induces the production of neutralizing antibodies at the same level as similar preparations based on E protein domain III of other flavivirus, but worse than whole-virion Tick-E-Vac vaccine. As a result, further refinement of this formulation is required so that it could be used for effective subunit TBE vaccine development.

One of the obvious ways to enhance the immunogenic potency of the preparation is to include other TBEV proteins [9] or additional fragments of TBEV E protein. This is especially important due to the fact that, as it was recently shown, the immune response to flavivirus infection differs between mouse and human [10, 32]. In particular, the specificity of neutralizing antibodies induced after TBEV infection or vaccination differs between these 2 species [10].

Another direction of improvement of the preparation is to optimize the adjuvant composition, since, as it is shown by Chiang [14], the adjuvant composition has a significant impact on the immunogenic potency and protectivity of formulation containing E protein domain III as antigen.

Another way to improve the immunogenic and protective properties of the preparation may be an optimization of immunization schedule, including the use of recombinant TBEV E protein domain III based formulation for booster immunization after immunization with whole-virion inactivated vaccine. It was shown that a booster injection of E protein domain III after immunization with inactivated virus or by soluble protein results in considerable enhancement of the neutralizing capacity of the serum in comparison with the double injection of inactivated virus or soluble E protein [13]. Thus, the optimization of immunization schedule can prolong the immunity response against TBE and decrease the number of re-vaccinations that would lower the costs of re-vaccinations.

## Conclusions

All 3 recombinant proteins comprising 3 variants of TBEV E protein domain III immobilized on dextran can specifically interact with the sera of patients with TBE. Thus, constructed recombinant proteins are promising for TBE diagnostics. It is also shown that the preparation comprising the recombinant TBEV E protein domain III immobilized on dextran and CpG oligonucleotides induces the production of neutralizing antibodies, but demonstrates limited protectivity as compared with Tick-E-Vac vaccine and, thus, requires further optimization to enhance immunogenic properties and protective capability. Nevertheless, this approach could be used for new vaccines that could replace the existing vaccines at least on the stage of boost vaccination, if not entirely.

## Additional files

Additional file 1: Fasta contains a multiple alignment of E protein domain III of TBEV. (FASTA 75 kb)
Additional file 2: Table S1. With the ELISA and Western blot (WB) data and **Table S2.** with the clinical symptoms in mice, immunized by different schemes. (PDF 158 kb)

## Abbreviations

DBD2: Dextran binding domain DBD2 from *Leuconostoc citreum* KM20
DIII: TBEV E protein domain III
PRNT50: 50 % plaque reduction neutralization test
TBE: Tick-borne encephalitis
TBEV: Tick-borne encephalitis virus
