# Supplementary material for: Recombinant domains III of Tick-Borne Encephalitis Virus envelope protein in combination with dextran and CpGs induce immune response and partial protectiveness against TBE virus infection in mice
Source: BMC Infect Dis. 2016 Oct 7;16:544. doi: 10.1186/s12879-016-1884-5 (PMC5054610; doi:10.1186/s12879-016-1884-5)
Supplement: Additional file 2: Table S1. — With the ELISA and Western blot (WB) data and Table S2. with the clinical symptoms in mice, immunized by different schemes. (PDF 158 kb) [file 12879_2016_1884_MOESM2_ESM.pdf]

Supplemental Table 1. ELISA and Western blot (WB) analysis of recombinant proteins, immobilized on dextran, with sera of TBE patients or healthy donors.

# sample [a]	TBEV subtype prevalent in the region	IgG titer ELISA	DBD2-D3S	DBD2-D3E	DBD2-D3F	DBD
			ELISA/WB	ELISA/WB	ELISA/WB	WB
96	Siberian	1:2400	+/+ [b]	+/+	+/+	–
M 151	European, Siberian	1:200	+/+	+/+	+/+	–
375 (II)	Siberian	1:2800	+/+	+/+	+/+	–
402 (II)	Siberian	n/d [c]	+/+	–/+	+/+	–
M 988	Siberian	<1:100	–/+	–/+	–/+	–
M 2545	Siberian	1:400	+/+	+/+	+/+	–
M 2546	Siberian	1:1400	+/+	+/+	+/+	–
M 2548	Siberian	<1:100	+/+	+/+	+/+	–
M 2549	Siberian	1:1700	+/+	+/+	+/+	–
M 2550	Siberian	1:300	+/+	+/+	+/+	–
M 2551	Far-Eastern	1:1800	+/+	+/+	+/+	–
M 1311	Siberian	<1:100	–/–	–/–	–/–	–

[a] Serum of a healthy donor is shown in bold italic, all other sera samples are from patients with clinically proven TBE diagnosis.

[b] +- presence of effect (antibodies in ELISA, or band in WB); – - absence of effect (antibodies in ELISA, or band in WB)

[c] n/d – not done

Supplemental Table 2. Clinical symptoms in mice, immunized by different schemes.

Preparation	N mice	Day 0 1st dose	Preparation	Day 7 2nd dose	Day 14 3d dose	Day 21[b] i/p 200 LD_{50}	Day 42 N mice		Survived animals with clinical symptoms[c]	
							Survived	Healthy	mild (m=1)	severe (m=2)
Standard scheme										
3DIII+AD	10	+[a]	3DIII+AD	+	+	TBEV Vas	5	0	1	4
Prime-boost scheme										
Tick-E-Vac	10	+	3DIII+AD	+	+	TBEV Vas	10	6	4	0
Reference scheme										
Tick-E-Vac	9	–	Tick-E-Vac	+	+	TBEV Vas	9	9	0	0
Control group										
Saline	10	+	Saline	+	+	TBEV Vas	0	0	0	0

[a] + mice were vaccinated; – mice were not vaccinated

[b] – day of challenge

[c] m=1 if mice were untidy, clumsy, and lost weight over 1.5g for at least 3 days; m=2 if mice showed signs of intoxication, paresis and paralysis of limbs.
